# Response of Plant Secondary Metabolites to Environmental Factors

**DOI:** 10.3390/molecules23040762

**Published:** 2018-03-27

**Authors:** Li Yang, Kui-Shan Wen, Xiao Ruan, Ying-Xian Zhao, Feng Wei, Qiang Wang

**Affiliations:** Ningbo Institute of Technology, Zhejiang University, Ningbo 315100, China; yangli0817@yeah.net (L.Y.); kuishanwen@zju.edu.cn (K.-S.W.); ruanxiao@nit.net.cn (X.R.); zyx@nit.net.cn (Y.-X.Z.); weifeng@nit.net.cn (F.W.)

**Keywords:** plant secondary metabolites, phenolics, flavonoids, terpenoids, alkaloids, responses, environmental factors, light irradiation, temperature, soil water, soil fertility and salinity

## Abstract

Plant secondary metabolites (SMs) are not only a useful array of natural products but also an important part of plant defense system against pathogenic attacks and environmental stresses. With remarkable biological activities, plant SMs are increasingly used as medicine ingredients and food additives for therapeutic, aromatic and culinary purposes. Various genetic, ontogenic, morphogenetic and environmental factors can influence the biosynthesis and accumulation of SMs. According to the literature reports, for example, SMs accumulation is strongly dependent on a variety of environmental factors such as light, temperature, soil water, soil fertility and salinity, and for most plants, a change in an individual factor may alter the content of SMs even if other factors remain constant. Here, we review with emphasis how each of single factors to affect the accumulation of plant secondary metabolites, and conduct a comparative analysis of relevant natural products in the stressed and unstressed plants. Expectantly, this documentary review will outline a general picture of environmental factors responsible for fluctuation in plant SMs, provide a practical way to obtain consistent quality and high quantity of bioactive compounds in vegetation, and present some suggestions for future research and development.

## 1. Introduction

As distinguished from primary metabolism and first attributed to Kossel [1], plant secondary metabolism is defined as a term for pathway and small molecule products of metabolism that are non-essential for the survival of the organism. In nature, a variety of secondary metabolism pathways elicited an array of plant defensive compounds called secondary metabolites (SMs). In addition to basic nutrients such as proteins, fats or carbohydrates, plants can produce other compounds including taxoids, polysaccharides, flavones, etc. SMs are the molecules to be dispensable for plant metabolism and growth, whereas the wide variety and high diversity of secondary products are key components for plants to interact with the environment in the adaptation to both biotic and abiotic stress conditions [2,3]. In fact, secondary metabolites involved in the protection against herbivores, bacteria, fungi, viruses and even other competing plants. In addition, some plants made use of secondary metabolites as signals for communication between plants and symbiotic microorganisms, as well as served to attract pollinators and seed dispersers [3,4].

**Figure 1:**
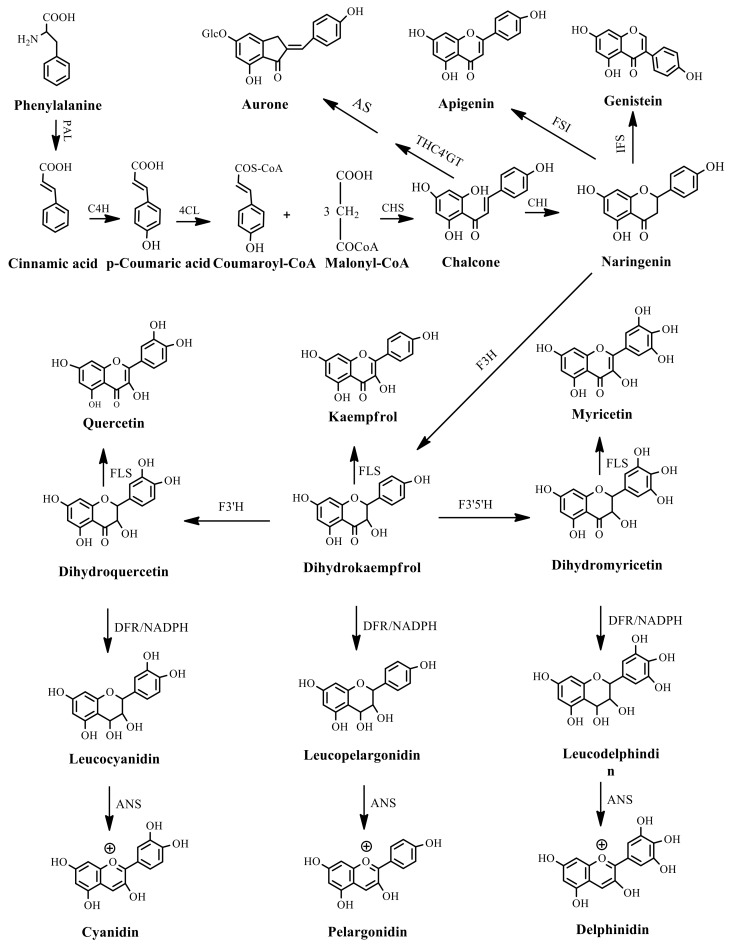
Classification and biosynthisis of flavonoids in plant (ANS = Anthocyanid in synthase; AS = Aureusidin synthase; C4H = Cinnamate-4-hydroxylase; CHI = Chalcone isomerase; 4CL = 4-coumaroyl: CoA-ligase; CHS = Chalcone synthase; DFR = Dihydroflavonol 4-reductase; F3H = Flavanone-3-hydroxylase; F3′H = Flavonoid 3′-hydroxylase; F3′5′H = Flavonoid 3′5′-hydroxylase; FLS = Flavonol synthase; FSI = Flavone synthase; IFS = Isoflavone synthase; PAL = Phenylalanine ammonia lyase; THC4′GT = UDP-glucose: tetrahydroxychalcone 4′GT).

**Figure 2:**
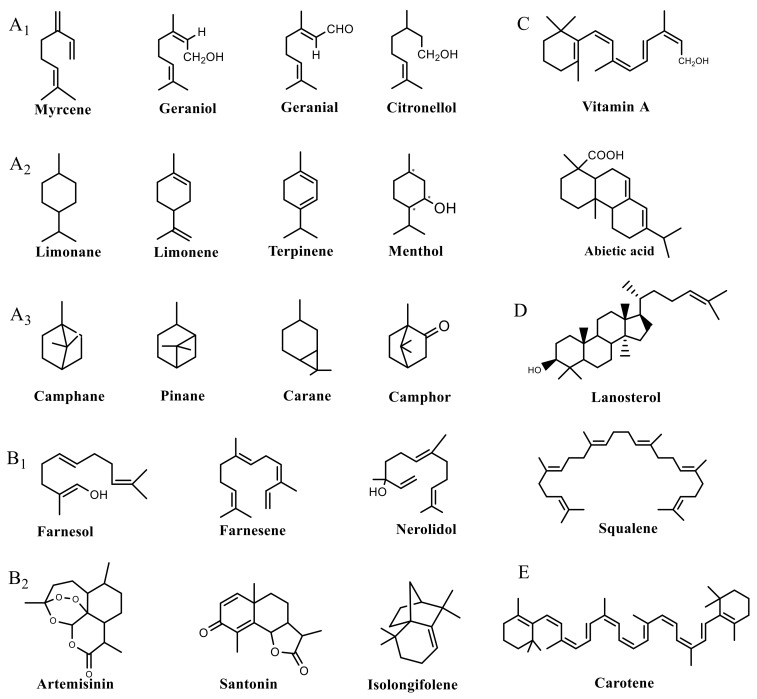
Classification of terpenoidsin plant. (**A**): monoterpenes, **A_1_**: Open-chain, **A_2_**: Single ring, **A_3_**: Bicyclic; (**B**): sesquiterpenes, **B_1_**: Open-chain, **B_2_**: Cyclic; (**C**): Diterpenes; (**D**): Triterpenes; (**E**): Tetraterpenes.

**Figure 3:**
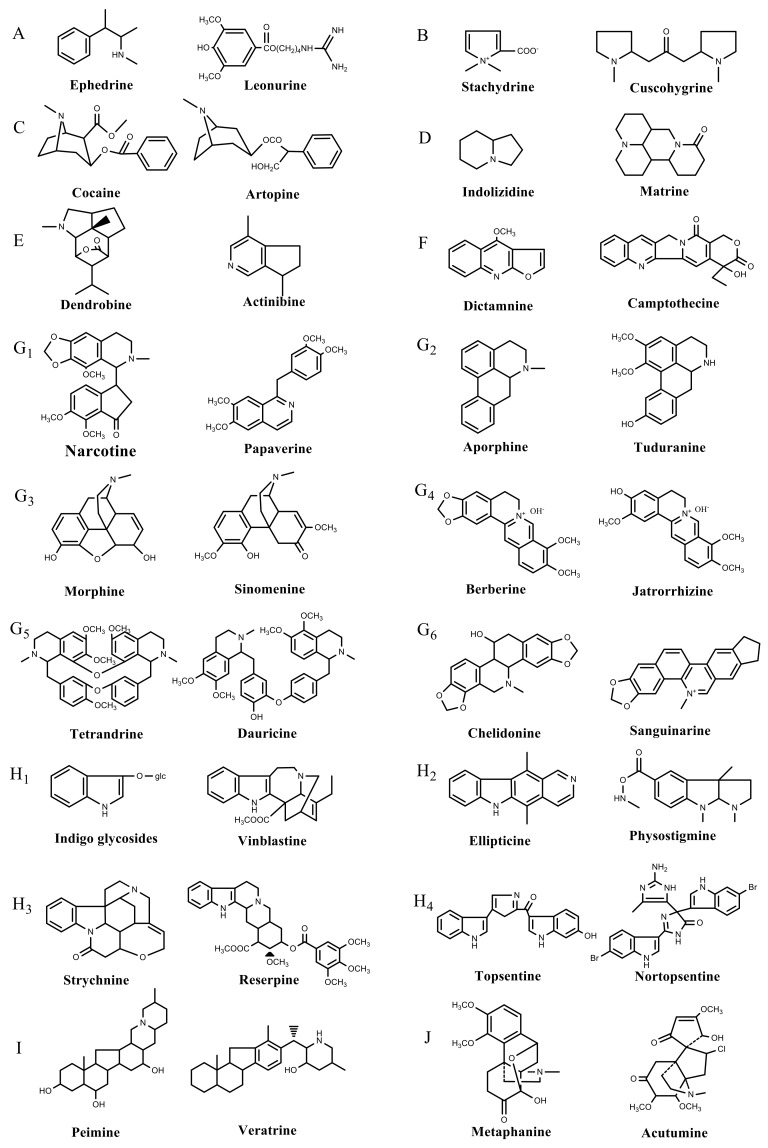
Classification of alkaloids in plant. (**A**): Amines; (**B**): Pyrrolidines; (**C**): Tropanes; (**D**): Piperidines; (**E**): Terpenoid; (**F**): Quinolines; (**G_1_**): 1-Benzylisoquinoline; (**G_2_**): Aporphines; (**G_3_**): Morphinane; (**G_4_**): Protoberberine; (**G_5_**): bisbenzylisoqunolines; (**G_6_**): Benzophenanthridines; (**H_1_**): Simple indoles; (**H_2_**): Tryptamine indoles; (**H_3_**): Monoterpenoid indoles; (**H_4_**): Bisindole alkaloids; (**I**): Steroidal alkaloids; (**J**): other alkaloids.

Plant SMs are usually classified according to their chemical structure [5]. Several groups of large molecules, including phenolic acids and flavonoids, terpenoids and steroids, and alkaloids have been implicated in activation and reinforcement of defense mechanisms in plants (see the classification and biosynthisis of flavonoids, alkaloids and terpenoids in plant at Figure 1, Figure 2 and Figure 3) [5,6]. Due to their remarkable biological activities, plant SMs have been formerly used as important source of active traditional medicine, perfume and industrial raw materials for centuries [7,8,9,10,11,12]. Whereafter, they were widely used as valuable compounds such as pharmaceutics, cosmetics, fine chemicals, or more recently nutraceuticals [13,14,15,16,17,18,19]. Obviously, SMs tremendously promoted to the commercial importance and value of plants.

However, the extraction and purification of SMs from wild natural plants normally require a complex and time consuming process, and even so, the yield of the desirable end-product is often very low [20,21,22,23]. Consequently, finding practical approaches to intensify the process and enhance the yield pose a serious challenge to researchers. Up to now, many studies on searching of the highest yield plant species and optimization of culture conditions have been conducted [24,25,26,27,28], but few directly emphasized on the adaptability of plant secondary metabolites in response to environmental disturbances and stimulations. In fact, synthesis and accumulation of phytochemical components critically depended on environmental conditions. For most plants, external factors or variables (light, temperature, soil water, soil fertility and salinity) can significantly affect some processes associated with growth and development of the plants, even their ability to synthesize secondary metabolites, eventually leading to the change of overall phytochemical profiles which play a strategic role in production of bioactive substances [29,30,31]. In other words, plant secondary metabolites can be gradually generated in response to environmental stress, and hence plant secondary metabolism be viewed as plant behavior that is in part the ability of adaptation and survival in response to environment stimuli during the lifetime [32], and serve to establish ecological relationships between plants and other organisms [33]. In particular, for medicinal plants, environmental conditions are capable of redirecting the metabolism to consequently regulate the production of active constituents. Because, plants as herbal medicines have a complex and variable chemical composition, the type and amount of SMs as well as biological effects were often determined according to the change of environment.

Besides, specific SMs are synthesized only in certain circumstances, and thus, high quality medicinal plants should be raised produced only under carefully controlled environments. There are extensive literature documents on plant secondary metabolites. Here, we will focus on discussing how the yield and content of SMs can be varied with the changes in environmental and soil nutrients and agroclimatic conditions. In the following sections, the adaptability of plant SMs to key environmental factors including light irradiation (duration, intensity, quality), temperature, soil water, soil fertility and salinity will be in turn reviewed. The primary purpose of this literature commentary is to enhance our understanding of plant SM adaptability in response to environmental stresses, and then optimize cultivation techniques or ambient conditions to maximize yield of beneficial plant SM natural products in an efficient and sustainable manner.

## 2. Response of Plant SMs to Light Irradiation

Light is indispensable to biosynthetic course of a growing plant. The key factors related light radiation include photoperiod (duration), intensity (quantity), direction and quality (frequency or wavelength) [34,35]. In the natural world, light plays an inreplaceable role in promoting plant growth and inducing or regulating plant metabolism. In response to light radiation, plants are able to adapt to the changes of circumstances by the release and accumulation of various secondary metabolites including phenolic compounds, triterpenoids and flavonoids, and many of them, have high economic and utilization value due to the well-known antioxidant property.

### 2.1. Effect of Photoperiod on Plant Secondary Metabolites

Photoperiod factor would influence the growth and development of plants, and thus regulate the biosynthesis of SMs [36,37]. Early studies showed that the duration of light radiation had a predominant role in regulating the levels of various phenolic phenylpropane derivatives in the *Xanthium* species. In comparison with a long day of light exposure, a short day of light exposure caused a decrease of, caffeoylquinic acids by about 40% and even an approximately double reduction in the content of flavonoid aglycones [38]. Similarly, grown under short-day treatment, the content of anthocyanins in *Pinus contorta* seedlings grown under short sunlight was notably lower than those growing in the long sunlight area, whereas the concentration of proanthocyanins and flavan-3-ols changed little as sunshine period varied [39]. A long period (16 h) of light irradiation on leaves of *Ipomoea batatas* generated a dramatic increase in the content of flavonoids (anthocyanins, catechins and flavonols) and phenolic acids (hydroxycinnamic and hydroxybenzoic acids) [40]. In a controllable photoperiod experiment, the analyses on chemical compositions of *Vaccinium myrtillus* from Northern (Lapinjarvi) and Southern (Oulu and Muhos) regions of Finland indicated that the southern clones produced the highest anthocyanin and its derivatives during a 24-h light period, and in comparison with a shorter photoperiod (12 h), longer photoperiod (24 h) also enchanced the level of chlorogenic acid [41]. In general, a long-day sunshine can increase the level of SMs in plants, and further support the role of flavonoids and phenolic acids in protecting plants to resist light exposure. The effect of photoperiod change on the content of plant secondary metabolites was illustrated in Table 1.

### 2.2. Effect of Light Intensity on Plant Secondary Metabolites

As a familiar indole alkaloid [42,43], the SM camptothecin can respond to environmental stresses and its accumulation rate can change with light irradiation conditions. It is known that overshadowing can induce biochemical changes in plants, particularly in leaves [44], and heavy shading of only 27% full sunlight, for example, can elevate the concentration of camptothecin in leaves of *Camptotheca acuminata*, whereas substantially reduce that in the lateral roots of this tree [45]. 

For *Centella asiatica* populations with high contents of secondary metabolites, qualitative and quantitative analyses show that light exposure can affect the content of bioactive triterpenes in these grasses, and under 70% shade-grown condition they contained the highest amount of asiatic acid but the lowest amounts of asiaticoside [46]. In addition, the content of flavonoids and chlorogenic acid in plants are positively correlated to the growth-lighting condition [47]. The exposure to full day sunlight resulted in an increase in contents of asiaticoside, madecassoside, flavonoids and chlorogenic acid in the plants compared with those grown under 50% of the shade [48]. These results indicate that the accumulation of triterpene and phenolic compounds in *C. asiatica* depends on duration and amount of daylight. The composition and antioxidant activities of phenolic compounds in *Berberis microphylla* were qualitatively and quantitatively evaluated under different light intensities. It is found that high light intensity can increase the content of monomeric anthocyanin by three times more than that medium light intensity does. For the content of total polyphenol, however, high light intensity only gives a slight increase compared with medium light intensity. In the meantime, high light intensity can activate the higher antioxidant capacity of SMs in plants [49]. The effect of light intensity on the content of plant secondary metabolites is shown in Table 2.

### 2.3. Effect of Light Quality on Plant Secondary Metabolites

Light quality can also influence synthesis of bioactive compound and secondary metabolism of plants. Monochromatic light was more sensitive than combined light to improve antioxidant phenolic compound of *Lactuca sativa* ‘Sunmang’. The total content of antioxidant phenols decreased with increasing the proportion of red light, and each of individual phenolic compounds including chlorogenic, caffeic, chicoric and ferulic acids as well as kaempferol showed a similar behavior [50]. UV irradiation is such an important abiotic factor that in many cases stimulates the production of secondary metabolites, and hence has been applied in cell and callus culture [24,51,52]. When the cells were irradiated with UV-B, the production of catharanthine and vindoline from *Catharanthus roseus* was enhanced [53]. The inducing effect of a continuous UV irradiation on the anthocyanin content of *Daucus carota* cell cultures was investigated and revealed that the total accumulation of anthocyanin was significantly enhanced by the UV irradiation [54]. As one of the major components, flavonoids were isolated from the *Cistus* exudates [55,56], whereas among various environmental and physical variables, the UV irradiation was considered to be the major inducer for the enhancement of flavonoids content [56]. UV irradiation on calluses of several *Passiflora* species was able to increase the production of all four glycosyl flavonoids [24]. Regvar et al. [57] comparatively evaluated the effect of UV irradiation on the concentration of rutin, catechin and quercetin in *Fagopyrum esculentum* and *F. tataricum*, and found a specific increase of quercetin concentration in *F. esculentum* when exposed to the enhanced UV irradiation. Warren et al. [58] found that the levels of flavonoids (mainly kaempferol and quercetin) in *Populus trichocarpa* leaves increased in response to UV-B irradiation. Markham et al. [59] compared C-glycosylflavones content of different rice cultivar under UV-B light, and found that C-glycosylflavones were enhanced in a UV-tolerant rice cultivar but absent in a susceptible cultivar. With supplemental UV-B, significant differences in flavonols accumulation were found among *T. repens* populations, and quercetin glycoside levels increased by 200% on average [60]. For *Hypericum perforatum*, a strong correlation between rutin content and the altitude of the grown site was observed. The amount of rutin in the plants grown at an altitude of 800 m was 4-fold higher than that in the plants grown at an altitude of 200 m, and this difference was attributed to discrepancy of solar radiation levels [61]. Therefore, the adjustment of light quality at a specific growth stage should be considered as a strategic tool for improving yield of SMs. The effect of light quality on the content of plant secondary metabolites is displayed in Table 3.

## 3. Response of Plant SMs to Temperature

During the 20th century, the average global temperature has already increased by 0.74 °C, approximately increased by 0.2 °C per decade [62]. According to recent global climate models, the annual mean temperature in Finland and Northern Europe is predicted to be 1.2–1.5 °C higher by year 2040 [62,63]. Authoritative simulation estimates that climate warming will cause a considerable affection on the production of SMs from vegetation. 

As one of the major weather variables temperature can significantly influence the composition of SMs, and in general raising temperature might almost enhance all of SMs in plant species. For instance, the composition of phenolic compounds (delphinidin-3-*O*-glucoside, delphinidin-3-*O*-rutinoside, and myricetin-3-*O*-glucoside) in three *Ribes nigrum* cultivars demonstrated the positive correlations with temperature [64], providing the important guidelines for berry cultivation for commercial exploitation. 

The modulation of temperature to alkaloids accumulation was reported, and high temperature preferable to induce the biosynthesis of alkaloids. The total accumulation of alkaloids (morphinane, phthalisoquinoline and benzylisoquinoline) in dry *Papaver somniferum* was restricted at low temperature [65]. In contrast, the total level of phenolic acids and isoflavonoid (genistein, daidzein and genistin) in soybean (*Glycine max*) roots increased after the treatment at low temperature for 24 h, and among which the highest increase of about 310% was observed in genistin after the treatment at 10 °C for 24 h, in comparison to the control [66].

High temperature incubation led to an instinct rise of 10-hydroxycamptothecin (HCPT) so that a 6-fold accumulation of HCPT in leaves of *C. acuminata* seedling occurred after the incubation at 40 °C for 2 h [67], indicating HCPT was involved in the defence against heat shock from the environment. For four consecutive years, higher alkaloid content in six cultivars of *Lupinus angustifolius* grown in field conditions was detected in 2006 with higher ambient temperature than other years [68]. Additional experiment in green house at different temperatures (10, 20 and 30 °C) also confirmed that higher temperature resulted in a higher alkaloid content [68]. The investigation on temperature affecting content of alkaloid in 60-day-old *C. roseus* seedlings showed that under short-term heat shock, the contents of vindoline, catharanthine and vinblastine in the seedling leaves were higher at 40 °C than those at 30 °C. More specifically, catharanthine content was increased by 40% after incubation at 40 °C for 2 h, while increased slowly at 30 °C and reached the highest value at 6 h, and in a long-term experiment at 35 °C, the concentrations of monomeric alkaloids catharanthine and vindoline showed a sharp increase [69]. When *C. roseus* leaves were exposed to low temperature, nearly 2-fold and 2–4 folds reduction in levels of catharanthine and vindoline were observed, respectively [70]. Besides, the concentrations of total piperidine alkaloids and two individual piperidine alkaloids in needles of 1-year-old Norway spruce exposed to high temperature were significantly higher than those in the needles under ambient temperature. Moreover, elevating temperature resulted in a decrease in the amounts of total flavonoids in bark as well as total catechins and total acetophenones in needles [71]. Overall, the regulation of alkaloids metabolism suggested that low temperature attenuated the regulation of most alkaloids biosynthetic pathway genes [70].

In recent years, the correlation between terpenoid yield and temperature were investigated for deciduous and coniferous vegetation species. The capacity for isoprene emission of *Quercus rubra* and *Q. alba* in warm conditions was twice that in cold conditions [72]. Analysis of most terpenes in *D. carota* root showed increasing values with increasing temperature, except that only α-terpinolene decreased significantly with increasing temperature [73]. In *D. carota*, high content of terpenes can initiate a strong bitterness to be unpleasant to the consumers, and thus it was necessary to avoid the high growth temperature for the tasty carrots. The emission of sesquiterpene compounds (SQTs) from seven pine species had a strong temperature dependency, in which the emission of β-caryophyllene, α-bergamotene, α-farnesene, and β-farnesene increased exponentially with temperature [74]. Also, the influence of night-time warming on the emission rates of volatile organic compounds (VOC) in birch (*Betula pendula*) and aspen (*P. tremula*) was investigated. In the case of *B. pendula*, the emissions of the C11 homoterpene 4,8-dimethy1-nona-1,3,7-triene (DMNT) and several sesquiterpenes except 3,7-guaiadiene were consistently increased with increasing night-time temperature (6–22 °C); nearly 100-fold increase in DMNT level was estimated in the birch exposed to 22 °C compared with that exposed to 6 °C; and total SQT emissions showed a very significant increase in higher temperatures of 18–22 °C. In the case of *P. tremula*, both DMNT and non-terpenes (NTs) were consistently increased from 6 to 14 °C, the total SQT emissions showed significant increase in the range of 6–18 °C, and the emission of total monoterpene and sesquiterpene reached a peak value at 18 °C [75]. In addition, an exponential increase with increasing temperature was observed in the emissions of many oxygenated monoterpenes except (*E*)-β-farnesene in Norway spruce (*Picea abies*), and besides there was no temperature effect on the total terpenoid emissions [76,77].

Elevation of temperature has been also confirmed to reduce the concentration of SMs in plants. Anthocyanin content in leaf sheaths of *Zea mays* seedlings increased with the severity and duration of cold, due to the induction of anthocyanin biosynthetic pathway genes [78]. Similarly, low temperature induced anthocyanin accumulation in leaves and stems of *Arabidopsis thaliana*, and facilitated anthocyanin synthesis through the phenylpropanoid pathway associated with increased transcripts of flavonoid biosynthetic genes including phenylalanine ammonialyase (PAL) and chalcone synthase (CHS) [79]. 

On the contrary, high temperature (35 °C) reduced the total anthocyanin content of *Vitis vinifera* cv. Cabernet Sauvignon to less than half of that in the control berries (25 °C), as a result of anthocyanin degradation and the inhibition of anthocyanin biosynthetic genes transcription [80]. Moreover, in various plants such as *Petunia hybrid* [81], *Citrus sinensis* [82], *Rosa hybrida* [83], the anthocyanin accumulation was induced by low temperature and inhibited by high temperature. The effect of temperature on the content of plant secondary metabolites was shown in Table 4.

## 4. Response of Plant SMs to Soil Water

Water stress is one of the most important environmental stresses that can regulate the morphological growth and development of plants, and alter their biochemical properties [84,85]. Severe water deficit has been considered to reduce the plants growth, but several studies have demonstrated that water stress may be possible to increase the amount of SMs in a wide variety of plant species.

In a wide range of experiments, it was indicated that when exposed to drought stress plants indeed accumulate higher concentrations of SMs. Nogués et al. [86] showed that for *Pisum sativum* cv. Meteor, the concentration of flavonoid in the plants suffered drought was increased by 45%, and also anthocyanin significantly increased by drought stress compared with the well-watered controls. In *Rhodiola sachalinensis*, maximal yield and content of salidroside were reached under the relative soil moisture of 55–75% [87]. Due to the vasoactive properties of *Crataegus*, its polyphenolic constituents have been attracting more and more attention. Two species of *Crataegus* (*C. laevigata* and *C. monogyna*) were subjected to water deficit (continuous water deprivation) and surplus (roots immersed in water) circumstances to assess the effects of water stress on levels of polyphenolics in them, which revealed that deficient water stress would induce the increases in contents of chlorogenic acid, catechin, and (−)-epicatechin, but superfluous water cause no net increases in the contents and in some cases, even a decrease in levels of polyphenolics [88]. Castellarin et al. [89] reported that the biosynthesis of anthocyanin in ripening fruit was strongly up-regulated by drought stress. Water deficit can also enhance the production of flavonoids in cell suspension culture of *Glycyrrhiza inflata* Batal [90]. The effect of drought stress on the concentration of active compounds in roots of *Salvia miltiorrhiza* was analyzed by Liu et al. [91], who found a massive increase in all analyzed compounds except for rosmarinic acid, and among them, the levels of tanshinones increased at a most in severe drought (SD) condition, including that the tanshinone I increased by 182%, and tanshinone IIA even increased by 322% in comparison with an increase of 148% in medium drought (MD) situation. Similar to tanshinone, the increase trend of cryptotanshinone due to SD stress was also observed. In recent years, several studies have focused on the molecular mechanism of secondary metabolism in response to abiotic stresses. The activation of genes involved in secondary metabolism and biosynthesis of phenolics. For example, Lettuce (*L. sativa*) plants through water stress can activate PAL gene participating in the biosynthesis of various phenolics and flavonoids [92]. Yuan et al. [93] reported that water deficit enhanced the expression of several flavonoids biosynthesis genes in *Scutellaria baicalensis* Georigi roots. Over-expression of *AmDEL* gene from *Antirrhinum majus*, resulting in both peel and flesh of fruit with intense purple colouration, significantly increased flavonoids accumulation [94]. The over-expression of *AmDEL* to *Arabidopsis* plants and WT plants were incubated for 4 weeks under drought stress to evaluate the plant response, indicating that the content of total flavonoids was significantly increased in the tolerant transgenic plants compared to WT plants [95].

As an indole alkaloid to be concentrated in seedlings of *C. acuminate*, camptothecin was inducible by progressive drought stress [96]. The influence of drought stress on the content of alkaloid in *P. somniferum* was determined after 5 days of cutting off water supply. The comparison to the control group demonstrated that three sorts of alkaloids (narkotine, morphine, codeine) emerged, and the peaks of narkotine and morphine became higher after a short period [97]. As compared with the unstressed control plants, the accumulation of total alkaloids in both shoot and root of *C. roseus* significantly increased under the oxidative stress due to drought [98]. The content of glycine betaine (GB) in *C. roseus* plants increased due to drought, because the drought stress can induce GB synthesis by over-expression of betaine aldehyde dehydrogenase, suggesting that this osmolyte played an important role in protecting plant cell mechanism under condition of drought [98].

In conifer species *P. sylvestris* and *P. abies*, total monoterpenes and resin acids in the seedlings of *P. sylvestris* suffering from severe drought were 39% and 32% higher than the controls, and they in *P. abies* seedlings experiencing severe drought were 35% and 45% higher [99]. Up to now, however, the few studies have been carried out on the effect of abiotic stresses on primary and secondary metabolism of *S. officinalis*. Among them, only two investigations have involved the effect of water deficit on this species. In the first study, Bettaieb et al. [100] reported the effect of drought on fatty acids and essential oil composition, and in the second one, Munné-Bosch et al. [101] evaluated the effect of this stress on diterpenes and tocopherols. *S. officinalis* under 70% of the optimal water supply revealed the contents of monoterpenes about 33% higher than those of plants cultivated under wellwatered conditions [102]. Moreover, the amount of terpenoidphytoalexins in roots of maize (*Z. mays*) experiencing the decline of subterranean volumetric water content (VWC) and salt stress was analyzed. In general, higher quantities of zealexins and kauralexins were contained in roots exposed to lower water content. The root tissues stressed by 30% VWC had zealexins 4-fold more than those of the control with 60% VWC. And the kauralexins content significantly increased by 2.2-fold under more modest drought conditions [103]. Correspondingly, the quantity of phytoalexins gradually accumulated as drought day continued, but plant vigour appeared to descend throughout the time course. After 5 days of drought (one-third of the VWC remained), the contents of both zealexin and kauralexin were significantly induced to elevate by three- and four-fold respectively, and continued to rise as drought persisted [103]. In a study of *C. asiatica*, the variation of asiaticoside and madecassoside contents were reported for different environmental conditions. As humidity and temperature elevated, samples exhibited the highest content of both asiaticoside and total triterpenes [104]. Another study reported the content of asiaticoside was higher during the rainy season and lower during the dry season [105]. More vividly, the effect of soil water on the content of plant secondary metabolites is displayed in Table 5. 

## 5. Response of Plant SMs to Soil Salinity

As one of the most brutal abiotic stresses, salt stress restricted the profitable production of natural products. In the world-wide, the area of the highly salinized lands has been increased to exceed 800 million hectares [106]. Salinization can induce complex interactions among various morphological, physiological and biochemical processes [107,108,109]. Also, Salinization may cause the oxidative stress due to high production of reactive oxygen species (ROS) so as to alter plant metabolism. In fact, plants produce a large number of SMs to scaveng or detoxify ROS.

In duration of *Aegiceras corniculatum* treated by 250 mM NaCl, polyphenol content significantly increased more than double as compared to control plants, suggesting that the accumulation of polyphenols played a role as protective metabolites [110]. In two Tunisian accessions of *Cakile maritima* (Jerba and Tabarka), the accumulation of polyphenols in Jerba was significantly increased by 56% and 30% in response to the treatment of 100 mM and 400 mM NaCl respectively, while that in Tabarka declined due to the NaCl treament [111]. After the treatment with moderate salinity (25–50 mM NaCl), the phenolic content in leaf of *Cynara cardunculus* was dramatically enhanced to reach the peak value corresponding to NaCl concentration of 50 mM [112]. The stresses of various salinity resulted in the accumulation of phenolic compounds in *F. esculentum* to be 57%, 121% and 153%, higher than that of the control treated with 10, 50, and 100 mM for 7 d, respectively. Moreover, the accumulation of phenolic compounds was primarily caused by an increase in the contents of four major compounds including isoorientin, orientin, rutin and vitexin [113]. The increasing salinity was also found to stimulate the biosynthesis of phenols and oleuropein in four olive cultivars, especially in leaves. The increase of total phenols content was abrupt at 125 mM NaCl which was more than double to that of control plants occurred in all cultivars. Due to the highest salinity treatment the concentration of Oleuropein was 18.5, 5.5, 2.5 and 3.8 folds greater than those of the control plants for ‘Zard’, ‘Ascolana’, ‘Koroneiki’ and ‘Arbequina’, respectively. However, the variation trend of leaf hydroxytyrosol concentration was different from that of oleuropein. When exposed to 125 mM NaCl, the hydroxytyrosol in each one of the all cultivars decreased abruptly below the values of the control plants [114]. Among three chloride salts (NaCl, KCl and CaCl_2_), KCltreatment showed a more pronounced effect on the contents of total phenolics and flavonoids in leaves of artichoke (*C. cardunculus*) and cardoon (*C. cardunculus* var. *altilis*) [115]. Moreover, the rapeseed (*Brassica napus* var *oleifera*) under increasing salinity was sprouted to evaluate the effect of salinity on total phenolics (TP), non-flavonoids (NF), tannins (TAN), phenolic acids (PAs). In early sprouts, TP increased by 35% with salinity up to 50 mM NaCl, as compared with control and then decreased slightly, the maximum increase of total-NF (30%) was showed in corresponding to the treatment of 25 mM NaCl, and total-TAN increased with salt concentration up to 50 mM and remained such high in response to the treatment of 100 and 200 mM NaCl, while salinity did not give a clear effect on total-Pas content. Overall, a moderate salinity in 25–50 mM NaCl caused the highest relative increase in phenolic concentration [116]. However, the accumulation of phenolic compounds in plants by salinity stress would also depend on the plant species, so that phenolic compounds failed to be accumulated in some plant species. With respect to the control plants, salinity stressed also led to a decrease of phenolic compounds (chlorogenic and sinapic acid derivatives and flavonoids) in leaves of broccoli (*B. oleracea* var. *italica* cv. Marathon) and the loss was higher for flavonoids than for sinapic acid derivates [117]. Furthermore, salinity stress can change the chemical contents of various phenolic compounds in rice cultivars (tolerant and susceptible varieties), causing a large increase in total phenolics and the content of vanillin and protocatechuic acid in tolerant varieties, whereas in contrast, a markedly reduce is found in the susceptible cultivar [118]. The effect of NaCl concentration on total phenolic content in *S. macrosiphon* showed that all the treatments with different concentration of NaCl elicited a remarkable reduction of total phenolic content in the leaf which decreased with the treatment of increasing NaCl concentration. After induced with 6.8 dS m^−1^ NaCl, the content of total phenolics was reduced by 2.6 times as compared to control leaves [118]. However, NaCl salinity increased total antioxidant activity in methanolic extract of the leaf, probably due to the increasing activity of peroxidase (POD) under salt stress conditions [119].

Jaleel et al. [98] reported that the content of indole alkaloidin *C. roseus* increased due to the treatment of 80 mM NaCl as compared to unstressed control plants. After *C. roseus* treated with 150 mM NaCl for 2 months, the content of vincristine in this plant significantly increased as compared with the control sample, but declined with further increasing salinity in a long-term treatment [120]. The yields of alkaloids in *C. roseus* increased gradually with the duration of seawater stress, and the plants treated by 5% seawater gave the yields of alkaloids higher than those treated by 10% seawater. Among the four kinds of alkaloids, the concentration of vindoline, catharanthine and vincristine in the plants treated by 5% seawater significantly increased as compared to the control. In consideration of industrial production, the treatments using 5% seawater can potentially reduce the cost of producing alkaloids [121]. In the medium of salinity equivalent to 100 mM NaCl, the accumulation of total alkaloids exceeded over that of non-saline control, and found to be maximal in roots of *C. roseus* [122].

Similarly, salinization can significantly alter the accumulation of secondary metabolite of rosemary (*Rosmarinus officinalis*), mainly inducing a pronounced effect on monoterpenes composition. It was founded that the solution of NaCl at 100 mM considerably increased the relative abundance of cineole and camphor, but slightly decreased those of borneol, α-terpineol, nopol, and camphene [123]. Moreover, the root tissues of maize exposed to salt stress can also increase the concentrations of acidic terpenoid phytoalexins, such that the immersion of the root tissues in the solution of NaCl at 500 mM dramedically enhance the quantity of zealexins by about five fold, while the treatment with lower concentration solution of NaCl (100 mM) significantly induce the content of kauralexins to raise by twofold in comparison with the control plants in the medium of 0 mM NaCl [103]. The effect of salinity stress on the content of plant secondary metabolites is exhibited in Table 6.

## 6. Response of Plant SMs to Soil Fertility

Supplemental plant mineral nutrition may provide a means not only to stimulate plant growth but also influence the content of SMs. Increasing studies have disclosed that the availability of plant nutrients can be an important factor in determining secondary metabolism and antioxidant activity within plants. Unfortunately, it should be mentioned that most fertilizers used in these studies were generally highly water-soluble simple fertilizers consisting of only one or two elements of N, P, or K, and therefore, inconsistent results have been reported. 

A large number of experiments have been proposed that nutrient deficiencies of plants is characterized by an accumulation of flavonoids, notably the anthocyanins. The inverse relationship between the availability of both nitrogen and phosphate availability and the content of flavonol in *Arabidopsis* and tomato seedling tissues was noted to be highly significant, and the concentration of quercetin, kaempferol and isorhamnetin was increased in response to either nitrogen or phosphate stress in both species [124]. Ibrahim et al. [125] found that nitrogen levels had a significant impact on the production of total phenolics and flavonoids in *Labisia pumila* Benth. As more nitrogen was invested steadily from 0 to 270 kg N ha^−1^ soil, less amount of phenolics and flavonoids was produced. The fertilizer at ratio of N:P:K = 1:0.6:1.2 can reduce the flavonoid content but increased polyphenol content in *B. microphylla*. Moreover, the antioxidant activities were slightly increased with the increment of fertilizing amount [49]. In the leaves of tomato (*Lycopersicon esculentum*) with N-dedicient rhizosphere condition, anthocyanins and one of flavonols consistently increased by 2- to 3-fold, while total non-anthocyanin flavonoids increased by 14% only, comparable to wild type plants. In addition, N-deficiency stress can also induce different effects on expression of genes encoding flavonoid biosynthetic enzymes, so that mRNA levels for chalcone synthase (CHS) and dihydroflavonol-4-reductase (DFR) increased while mRNA levels of a chalcone isomerase—homologous band (CHI) decreased in response to N stress [126]. As compared with the availability of fertilization, only nitrogen-free fertilization allowed to obtain the highest total polyphenols content and can exert a positive anti-radical activity and allowed to obtain the highest content of total polyphenols, although were not accompanied by an increase in terms of the main phenolic compounds [127,128].

Nutrient deficiencies in soil can affect the alkaloid concentration in seed of lupin (*L. angustifolius*). Under the condition of severe potassium deficiency, the alkaloid concentrations in the seeds of sweet varieties of lupins drastically increased by 205%. Over the range of K content from 0 to 240 mg kg^−1^ soil, lupanine was the predominant alkaloid in sweet varieties, whereas 13-hydroxylupanine prevailed in bitter variety [129]. Conversely, nitrogenous fertilizers significantly increased the content of alkaloids by 9–17%, and the greatest increase was found when NH_4_NO_3_ was applied [130]. Similar pattern of alkaloids in response to nitrogen supplement was also initiated in *L. albus* [131]. Simultaneous application of magnesium and nitrogen decreased the content of alkaloids in seeds [130]. The effect of Sangral compound fertilizer at rates of 0–800 kg ha^−^^1^ on the content of alkaloid in *Datura innoxia* plants was also investigated, indicating that the content of total alkaloids content increased with increasing the fertilization rate, reached a peak value at 600 kg ha^−1^, and then decreased at 800 kg ha^−1^ [132].

Owing to the application of the high concentration of ammonium nitrate fertilizer, Douglas-fir (*Pseudotsuga menziesii*) showed the increasing of monoterpenoid concentrations [133]. Compared with the high fertilizing amount of 400 mg, the application of 200 mg N-fertilizer increased the concentration of monoterpenoid in *Thuja plicata* during the active growing season (August), and when plant growth began to subside by September, however, the high fertilizing amount generated higher monoterpenoid concentration [134] as additional nutrient sources shifted toward terpenoid synthesis [135]. More intuitively, the effect of soil fertility on the content of plant secondary metabolites is showi in Table 7.

## 7. Conclusions

Plant adaptability to environmental stresses is a widespread ecological behavior in Nature. As a phenotypic and explicit behavior, plant morphological adaptability to environment is relatively easy to observe and recognize, while as an intrinsic and implicit behavior, plant biochemical adaptability is relatively hard to discover and not fully understood yet. Based on the relevant literature reports and data, the above review focuses on the response of important plant secondary metabolites such as phenolics, flavonoids, terpenoids and alkaloids produced from various biochemical processes to crucial environmental stresses, including light irradiation, temperature, soil water, soil fertility and salinity, etc. 

Here, we have provided a great deal of evidence to demonstrate the diversified and changeable responses of various plant secondary metabolites to different environmental stresses, What’s more interesting is that the individual environmental stress can selectively enhance the content of several SMs in plants. Thus it can be deduced that the synthesis of some natural products can be altered by diverse abiotic factors. As well known, some SMs have found commercial applications as drugs, flavours, fragrances, insecticides, etc. Such fine chemicals are extracted and purified from plant materials, but their production is often dependent on the physiological and developmental stage of the plant, and normally their content is too low to meet the requirements in the market of health care products, so the optimization of plant growth conditions to improve the concentrations of high value SMs becomes crucial and essential. This documentary analysis has suggested a theoretical basis to obtain consistent and high levels of SMs from plants. Prospectively, the use of environmental stresses may provide a potential and profitable way to increase the accumulation of bioactive compounds, improve quality, and reduce over-harvesting pressures of medicinal plants. 

Despite this review focused on the adaptability of secondary plant products in response to each individual environmental factor, the actual synthesis and accumulation of various SMs were frequently induced and/or modulated by a number of environmental factors simultaneously [41,49,71,136,137,138]. In other words, an individual factor can generally interact with other factors (e.g., a high irradiation is frequently accompanied with elevated temperature and water deficiency) Thus, we must further investigate and understand a synergistic effects of multiple environmental factors on plant secondary metabolism.

## Figures and Tables

**Table 1:** Photoperiod change on the content of various plant SMs.

Metabolite Class	Metabolite Name	Structural Image	Environment Factor	Concentration Change	Plant Species
Phenols	Caffeoylquinic acids [38]	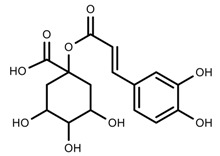	Short day of light	Decrease	*X. pensylvanicuim*
Phenols	Pelargonidin [39]	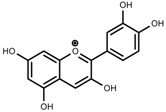	Short day of light	Decrease	*P. contorta*
Phenols	Catechins [40]	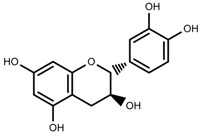	Long day of light	Increase	*I. batatas*
Phenols	Hydroxybenzoic acids [40]	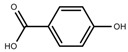	Long day of light	Increase	*I. batatas*
Phenols	Chlorogenic acid [41]	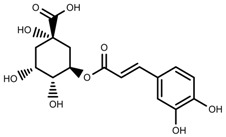	Long day of light	Increase	*V. myrtillus*

**Table 2:** Light intensity change on the content of various plant SMs.

Metabolite Class	Metabolite Name	Structural Image	Environment Factor	Concentration Change	Plant Species
Alkaloids	Camptothecin [45]	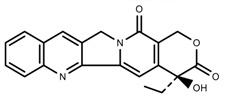	27% Full sunlight	Increase	*C. acuminate*
Phenols	Asiatic acid [46]	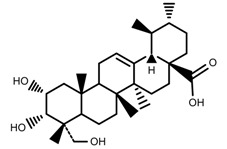	70% Shade	Increase	*C. asiatica*
Phenols	Asiaticoside [46]	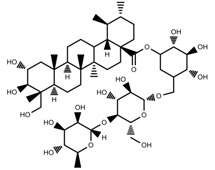	Full sunlight	Increase	*C. asiatica*
Phenols	Chlorogenic acid [47]	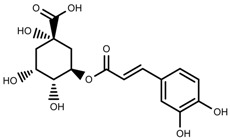	Full sunlight	Increase	*V. myrtillus*

**Table 3:** Light quality change on the content of various plant SMs.

Metabolite Class	Metabolite Name	Structural Image	Environment Factor	Concentration Change	Plant Species
Phenols	Ferulic acid [50]	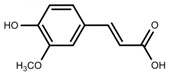	Increase red light	Decrease	*L. sativa*
Phenols	Kaempferol [50]	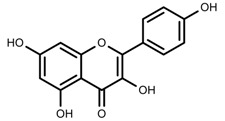	Increase red light	Decrease	*L. sativa*
Alkaloids	Catharanthine [53]	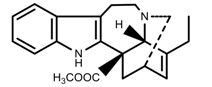	UV-B	Increase	*C. roseus*
Alkaloids	Vindoline [53]	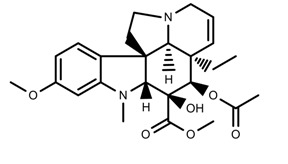	UV-B	Increase	*C. roseus*
Phenols	Rutin [57]	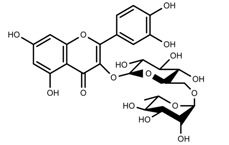	UV	Increase	*F. esculentum*
Phenols	Quercetin [57]	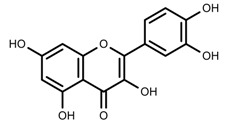	UV	Increase	*F. esculentum*
Phenols	Catechins [57]	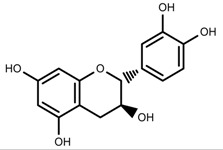	UV	Increase	*F. esculentum*

**Table 4:** Temperature change on the content of various plant SMs.

Metabolite Class	Metabolite Name	Structural Image	Environment Factor	Concentration Change	Plant Species
Alkaloids	Morphine [65]	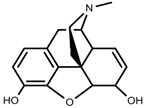	Low temperature	Decrease	*P. somniferum*
Phenols	Genistein [66]	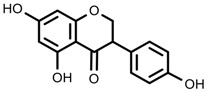	10 °C for 24 h	Increase	*G. max*
Phenols	Daidzein [66]	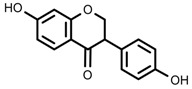	10 °C for 24 h	Increase	*G. max*
Alkaloids	10-hydroxycamptothecin [67]	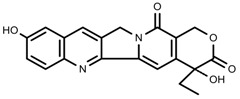	40 °C for 2 h	Increase	*C. acuminata*
Alkaloids	Vindoline [69]	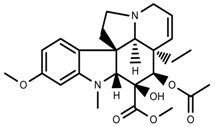	Short-term heat	Increase	*C. roseus*
Alkaloids	Catharanthine [69]	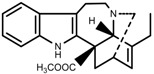	Long-term heat	Increase	*C. roseus*
Alkaloids	Vindoline [70]	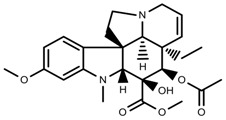	low temperature	Decrease	*C. roseus*
Terpenes	Isoprene [72]	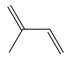	High temperature	Increase	*Q. rubra*
Terpenes	α-terpinolene [73]	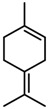	High temperature	Decrease	*D. carota*
Terpenes	β-caryophyllene [74]	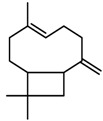	High temperature	Increase	*D. carota*
Terpenes	α-farnesene [74]	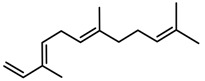	High temperature	Increase	*D. carota*
Terpenes	DMNT [78]	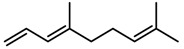	Night-time warming	Increase	*B. pendula*
Phenols	Pelargonidin [78]	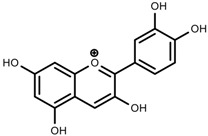	Low temperature	Increase	*Z. mays*

**Table 5:** Soil water change on the content of various plant SMs.

Metabolite Class	Metabolite Name	Structural Image	Environment Factor	Concentration Change	Plant Species
Phenols	Salidroside [87]	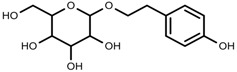	Soil moisture of 55–75%	Increase	*R. sachalinensis*
Phenols	Chlorogenic acid [88]	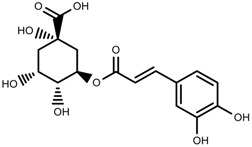	Deficit	Increase	*C. rataegus*
Phenols	Catechins [88]	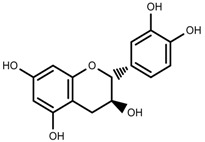	Deficit	Increase	*C. rataegus*
Phenols	(−)-epicatechins [88]	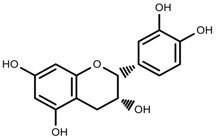	Deficit	Increase	*C. rataegus*
Phenols	Tanshinone [91]	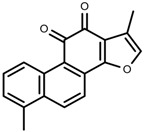	Severe drought	Increase	*S. miltiorrhiza*
Phenols	Cryptotanshinone [91]	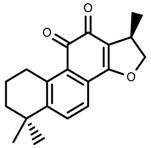	Severe drought	Increase	*S. miltiorrhiza*
Alkaloids	Camptothecin [96]	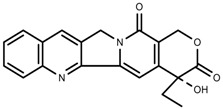	Drought	Increase	*C. acuminate*
Alkaloids	Morphine [97]	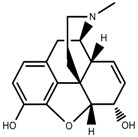	Drought	Increase	*P. somniferum*
Alkaloids	Codeine [97]	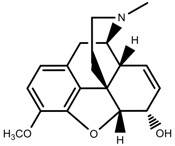	Drought	Increase	*P. somniferum*
Alkaloids	Glycine betaine [98]	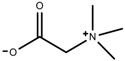	Drought	Increase	*C. roseus*
Phenols	Abietic acid [99]	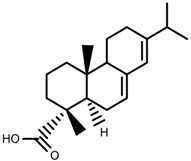	Severe drought	Increase	*P. sylvestris*
Phenols	Asiaticoside [104]	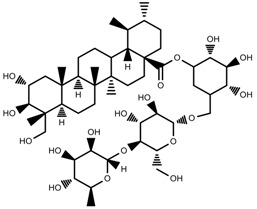	Humidity increase	Increase	*C. asiatica*

**Table 6:** Soil salinity change on the content of various plant SMs.

Metabolite Class	Metabolite Name	Structural Image	Environment Factor	Concentration Change	Plant Species
Phenols	Isoorientin [113]	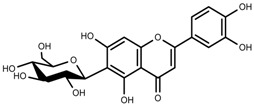	NaCl (10–100 mM)	Increase	*F. esculentum*
Phenols	Rutin [113]	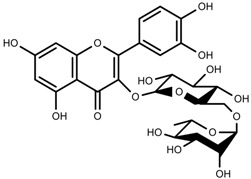	NaCl (10-100 mM)	Increase	*F. esculentum*
Phenols	Vitexin [113]	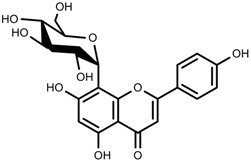	NaCl (10–100 mM)	Increase	*F. esculentum*
Terpenes	Oleuropein [114]	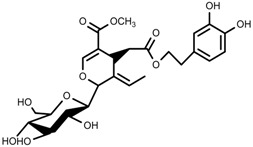	NaCl (125 mM)	Increase	*O. europaea*
Alkaloids	Catharanthine [121]	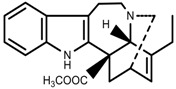	5% Seawater	Increase	*C. roseus*
Phenolic	Chlorogenic acid [117]	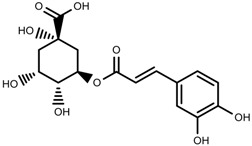	Salinity	Decrease	*B. oleracea* var. *italica* cv. Marathon
Phenolic	Vanillin [118]	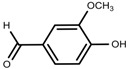	Salinity	Increase	*S. macrosiphon*
Phenolic	Sinapic acid [117]	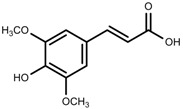	Salinity	Decrease	*B. oleracea* var. *italica* cv. Marathon
Phenolic	Protocatechuic acid [118]	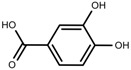	Salinity	Increase	*S. macrosiphon*
Terpenes	Borneol [123]	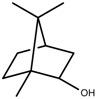	NaCl (100 mM)	Decrease	*R. officinalis*
Terpenes	Cineole [123]	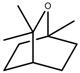	NaCl (100 mM)	Increase	*R. officinalis*
Terpenes	Camphene [123]	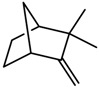	NaCl (100 mM)	Decrease	*R. officinalis*
Terpenes	Camphor [123]	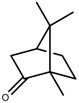	NaCl (100 mM)	Increase	*R. officinalis*
Terpenes	α-terpineol [123]	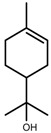	NaCl (100 mM)	Decrease	*R. officinalis*
Terpenes	Hydroxytyrosol [114]	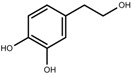	NaCl (125 mM)	Decrease	*O. europaea*

**Table 7:** Soil fertility change on the content of various plant SMs.

Metabolite Class	Metabolite Name	Structural Image	Environment Factor	Concentration Change	Plant Species
Phenols	Quercetin [124]	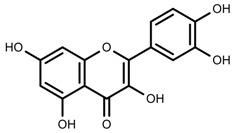	Nitrogen and phosphate	Increase	*L. esculentum* cv. Chaser
Phenols	Kaempferol [124]	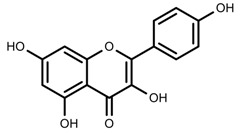	Nitrogen and phosphate	Increase	*L. esculentum* cv. Chaser
Phenols	Isorhamnetin [124]	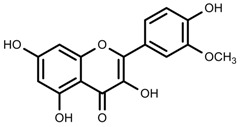	Nitrogen and phosphate	Increase	*L. esculentum* cv. Chaser
